# The influences of the M_2_R-GIRK4-RGS6 dependent parasympathetic pathway on electrophysiological properties of the mouse heart

**DOI:** 10.1371/journal.pone.0193798

**Published:** 2018-04-18

**Authors:** Kanchan Kulkarni, Xueyi Xie, Ezequiel Marron Fernandez de Velasco, Allison Anderson, Kirill A. Martemyanov, Kevin Wickman, Elena G. Tolkacheva

**Affiliations:** 1 Department of Biomedical Engineering, University of Minnesota, Minneapolis, Minnesota, United States of America; 2 Department of Pharmacology, University of Minnesota, Minneapolis, Minnesota, United States of America; 3 Department of Neuroscience, The Scripps Research Institute, Jupiter, Florida, United States of America; Universiteit Gent, BELGIUM

## Abstract

A large body of work has established the prominent roles of the atrial M_2_R-I_KACh_ signaling pathway, and the negative regulatory protein RGS6, in modulating critical aspects of parasympathetic influence on cardiac function, including pace-making, heart rate (HR) variability (HRV), and atrial arrhythmogenesis. Despite increasing evidence of its innervation of the ventricles, and the expression of M_2_R, I_KACh_ channel subunits, and RGS6 in ventricle, the effects of parasympathetic modulation on ventricular electrophysiology are less clear. The main objective of our study was to investigate the contribution of M_2_R-I_KACh_ signaling pathway elements in murine ventricular electrophysiology, using *in-vivo* ECG measurements, isolated whole-heart optical mapping and constitutive knockout mice lacking I_KACh_ (*Girk4*^*–/–*^) or RGS6 (*Rgs6*^*-/-*^). Consistent with previous findings, mice lacking GIRK4 exhibited diminished HR and HRV responses to the cholinergic agonist carbachol (CCh), and resistance to CCh-induced arrhythmic episodes. In line with its role as a negative regulator of atrial M_2_R-I_KACh_ signaling, loss of RGS6 correlated with a mild resting bradycardia, enhanced HR and HRV responses to CCh, and increased propensity for arrhythmic episodes. Interestingly, ventricles from mice lacking GIRK4 or RGS6 both exhibited increased action potential duration (APD) at baseline, and APD was prolonged by CCh across all genotypes. Similarly, CCh significantly increased the slope of APD restitution in all genotypes. There was no impact of genotype or CCh on either conduction velocity or heterogeneity. Our data suggests that altered parasympathetic signaling through the M_2_R-I_KACh_ pathway can affect ventricular electrophysiological properties distinct from its influence on atrial physiology.

## Introduction

The autonomic nervous system (ANS) plays an important role in regulating cardiac electrical activity. Both branches of the ANS, namely the sympathetic and parasympathetic systems, directly innervate the heart and influence cardiac electrophysiology. While stimulation of the sympathetic system increases cardiac contractility, heart rate (HR) and conductivity, stimulation of the parasympathetic system has an opposite effect. In many disease conditions, including hypertension and chronic heart failure, there is an imbalance between these two branches of the ANS [1–4].

Parasympathetic modulation of cardiac output is mediated primarily by acetylcholine (ACh) release from the vagus nerve which activates M_2_ muscarinic receptors (M_2_R). In sino-atrial nodal cells and atrial myocytes, the activation of M_2_R triggers the release of inhibitory G proteins, which then proceed to modulate the activity of multiple downstream targets. Among these is the activation of the G protein-gated inwardly rectifying K^+^ channel I_KACh_, a heterotetrameric complex formed by GIRK1 and GIRK4 subunits which exerts a prominent effect on both HR and HR variability (HRV) [5, 6]. *Girk4*^*–/–*^mice, which exhibit a complete loss of I_KACh_ activity, are less sensitive to carbachol (CCh)-induced bradycardia, show reduced HRV, and a resistance to pacing-induced atrial fibrillation [7]. Previous studies have also shown that M_2_R-I_KACh_ signaling is negatively regulated by regulator of G protein signaling (RGS) proteins [8–10]_._ In particular, a dimer consisting of the atypical G protein ß subunit Gß5 and RGS6, a member of the R7 RGS family of RGS proteins, has been shown to prominently accelerate I_KACh_ deactivation kinetics in sinoatrial nodal cells and atrial myocytes. Loss of RGS6 in mice results in an increase in I_KACh_ activity which, in contrast to *Girk4*^*–/–*^mice, correlates with an increase in CCh induced bradycardia, increase in HRV, and increase in susceptibility to pacing induced AF [8–10].

While the M_2_R-I_KACh_ signaling pathway is a critical mediator of parasympathetic influence on the atria, growing evidence suggests that a similar signaling pathway may exist in the ventricle [11]. For example, GIRK4 mRNA and protein are present, albeit at lower levels than in atria, in the ventricle of many species [8, 11, 12]. In addition, pharmacologic blockade or genetic ablation of GIRK4 correlates with diminished influence of ACh on action potential duration (APD) and resting membrane potential in rodent ventricular tissue. Similarly, RGS6 has been detected in the ventricle [8–10], however its impact on ventricular physiology is less well defined. Given the growing appreciation for parasympathetic innervation of the ventricles [13–16], there has been interest in understanding the molecular components which mediate parasympathetic input on ventricular physiology and arrhythmogenesis.

The main objective of our study was to gain deeper insight into the impact of parasympathetic signaling on ventricular electrophysiology. To this end, we used *in-vivo* ECG measurements and *ex-vivo* optical mapping to evaluate cardiac physiology in mice lacking GIRK4 or RGS6. Our findings show that RGS6 exerts a prominent influence on key supraventricular and ventricular physiological variables, and that its relationship with I_KACh_-dependent signaling likely differs between the atria and ventricle.

## Materials and methods

All experiments were approved by and done in accordance with the guidelines of the Institutional Animal Care and Use Committee at the University of Minnesota. *Girk4*^*-/-*^ and *Rgs6*^*-/-*^ lines were created as previously described [10, 17]. Mice were purchased from the Jackson Laboratory (Bar Harbor, ME) and housed at University of Minnesota's Research Animal Resources (RAR) facility. Mice were group-housed on a 12-h light/dark cycle, and given free access to food and water. Experiments were done on 2–3 month old mice of either gender. At the end of the experiments, animals were sacrificed by exsanguination while under anesthesia (ketamine/xylazine).

### In-vivo ECG measurements

Mice were anesthetized with ketamine and xylazine (35 and 5 mg/kg i.p., respectively) and ECG recordings were performed using IX-ECG12 recorder interfaced with Labscribe software (v2, iWorx Systems Inc., Dover, New Hampshire). After 20 min of stabilization period, 10 min of baseline ECG was recorded. Subsequently, 300 nM of CCh was administered via tail vein injection (*ex-vivo* EC_50_ = 0.2 uM [18]). The final concentration of CCh was estimated using approximated blood volume based on body weight of each mouse measured at the beginning of the experiment. CCh has been reported to have a relatively long half-life, and a duration of action lasting 4–8 hours in humans, with topical administration [19]. Hence, after a 5 min waiting period for the effect of CCh to reach equilibrium, 10 min of ECG was recorded. The mice were then sacrificed by exsanguination. The 10 min baseline and post-CCh intervals of ECG recordings were used to calculate HR and HRV, and to identify the occurrence of arrhythmic episodes induced by administration of CCh. Any noise artifacts were removed through data filtering and only clean segments of ECG data were used. A maximum HR threshold was set to 1000 bpm for baseline and mean baseline HR + 25 bpm for evaluating effect of CCh, in order to eliminate artifacts and erroneous spikes in HR. HRV was calculated as a ratio of standard deviation of HR to mean HR, as described previously [20]. Number of mice that exhibited arrhythmic episodes after injection of CCh were also quantified. An arrhythmic episode was defined as an occurrence of either bradycardia (at least 25 bpm < mean post CCh HR) or tachycardia (at least 25 bpm > post CCh HR) that lasted for more than a 2-s duration.

### *Ex-vivo* optical mapping experiments

Isolated *ex-vivo* whole heart optical mapping experiments were performed as described previously [21–23]. Briefly, mice were first heparinized (55 U/10g) to avoid blood clots during heart excision. Ketamine and xylazine (100 mg/kg and 10 mg/kg i.p., respectively) were administered to anesthetize the animal, after which hearts were quickly excised and immersed in cold cardioplegic solution (in mM): glucose 280, KCl 13.44, NaHCO_3_ 12.6, and mannitol 34. The aorta was isolated under microscope for cannulation and the hearts were retrogradely perfused using Langendorff perfusion setup with warm (37±1°C) Tyrode’s solution (in mM): NaCl 130, CaCl_2_ 1.8, KCl 4, MgCl_2_ 1.0, NaH_2_PO_4_ 1.2, NaHCO_3_ 24, glucose 5.5, and pH 7.4. After 15 min of stabilization, a bolus of the voltage-sensitive dye (di-4-ANEPPS, 5 μg/mL) was injected into the hearts. Two green lasers (532 nm, Shanghai Dream Lasers Tech., Shanghai, China) were used to excite the hearts and the fluorescence signal emitted was recorded from the left ventricular (LV) surface by a fast high-resolution (14-bit, 80x80 pixels, 1000 frames/s) CCD camera (Little Joe, RedShirt Imaging, SciMeasure). Mechanical uncoupler Blebbistatin (10 μM) was added to the perfusate to reduce motion artifacts when required. Background fluorescence was eliminated during analysis by using spatial (3 x 3 pixels) and temporal convolution filters.

The hearts were paced at progressively decreasing basic cycle lengths (BCL), starting from 150 ms down to 90 ms in steps of 10 ms. At each BCL, 40 stimuli were applied to reach steady state, and optical movies corresponding to the last 10 stimuli were recorded and later analyzed. After capturing baseline recordings, hearts were injected with a bolus of CCh, either 300 nM or 3 μM. After 10 min of stabilization, similar pacing protocol was repeated and optical movies were captured.

### *Ex-vivo* parameter measurements

Optical movies were analyzed to characterize the electrophysiological properties of the LV. The APD was calculated at 80% (APD_80_) repolarization. For each mouse, average of 5 action potentials was used to calculate mean APD_80_ at each pixel. Two dimensional (2D) APD maps were then constructed as described previously [21–23] to demonstrate the spatial distribution of APD. For each heart and at each BCL, average APD value for the entire LV surface was then calculated from individual pixel values. To investigate the dispersion of repolarization in LV, APD_80_ heterogeneity was calculated based on the heterogeneity index μ as,

$$\mu =(X^{95}-X^{5})/X^{50}$$

where X is APD_80_, X^95^ and X^5^ represent the 95^th^ and 5^th^ percentiles of the APD_80_ distribution, and X^50^ is the median of the APD_80_ distribution. Conduction velocity (CV) was calculated as described previously [23]. Specifically, the distributions of activation times measured at (dV/dt)_max_ for the spatial regions of 3 x 3 pixels were fitted with the plane, and gradients of activation times were calculated for each plane. The magnitude of the local CV was calculated for each pixel. APD restitution curves were generated by plotting mean APD_80_ as a function of mean diastolic interval (DI = BCL–APD_80_). The maximum slope of APD restitution, *S*_*max*_, was calculated by fitting with 2^nd^ degree polynomial and evaluating its derivative at the lowest DI value for each run. Mean *S*_*max*_ was then calculated for each genotype both at baseline and post administration of CCh.

### Statistics

All data are presented as mean ± standard error (SEM). Statistical comparisons were analyzed using 1-way ANOVA and Fisher’s exact test, as appropriate (Origin Software, Northampton, MA, USA). In addition, 2-way ANOVA was performed between genotype, CCh dosage and APD_80_ to investigate the role of GIRK4 or RGS6 in modulating the effects of CCh on APD prolongation. *P*<0.05 was the set level of significance.

## Results

### *In-vivo* effect of parasympathetic modulation on HR and HRV

**Fig 1:**
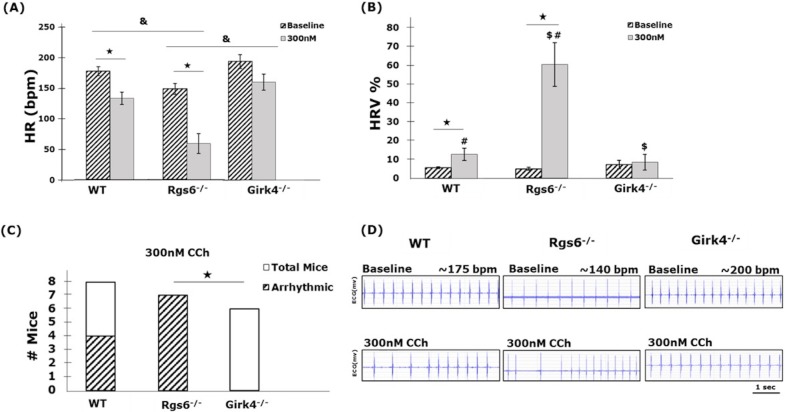
The influence of M_2_R-I_KACh_ signaling on in vivo HR and HRV. Summary of baseline and post-CCh (300nM CCh) in-vivo HR (A) and HRV (B) in WT, *Rgs6*^*-/-*^, and *Girk4*^*-/-*^ mice. (‘*’ denotes statistical significance of p < 0.05 between baseline and CCh within the same genotype. ‘&’ denotes a statistically significant (p < 0.05) difference for both baseline and CCh when comparing between two genotypes). ‘#’ denotes statistical significance of p < 0.05 between WT and *Rgs6*^*-/-*^ mice post-CCh. ‘$’ denotes statistical significance of p < 0.05 between *Girk4*^*-/-*^ and *Rgs6*^*-/-*^ mice post-CCh. Statistics performed using 1-way ANOVA.) (C) Quantification of the total number of mice that exhibited arrhythmias post CCh. (‘*’ denotes statistical significance of p < 0.05 between *Girk4*^*-/-*^ and *Rgs6*^*-/-*^. Statistics performed using Fisher’s exact test). (D) Representative examples of ECG data during control and demonstrating episodes of arrhythmia in WT and *Rgs6*^*-/-*^, and no arrhythmia in *Girk4*^*-/-*^ mice post CCh. n = 8, 8, 6 for WT, *Rgs6*^*-/*-^, and *Girk4*^*-/-*^_,_ respectively.

To confirm the impact of M_2_R-I_KACh_ signaling on atrial physiology, HR and HRV was assessed in WT, *Girk4*^*–/–*^, and *Rgs6*^*–/–*^mice at baseline and after CCh injection using *in-vivo* ECG recordings from anesthetized mice. Consistent with previous reports, *Rgs6*^*-/-*^ mice exhibited significantly lower baseline HR compared to WT mice (Fig 1A). Conversely, *Girk4*^*-/-*^ mice displayed slightly higher baseline HR, however, this effect was not significant (Fig 1A). Injection of CCh evoked a significant decrease in HR for both WT and *Rgs6*^*-/-*^ mice, whereas no significant change in HR was seen for *Girk4*^*-/-*^ mice (Fig 1A). Furthermore, CCh significantly increased HRV in both WT and *Rgs6*^*-/-*^ mice, while this effect was absent in *Girk4*^*-/-*^ mice (Fig 1B). In addition, *Girk4*^*-/-*^ mice were resistant to the onset of arrhythmias post injection of CCh, as opposed to both *Rgs6*^*-/-*^ and WT mice (Fig 1C). These results confirm previously reported phenotypes describing the prominent role the M_2_R-I_KACh_ signaling pathway plays in mediating atrial physiology and how RGS6 is a critical regulator of its influence.

### *Ex-vivo* optical mapping assessment of M_2_R-I_KACh_ signaling in the mouse ventricle

While the atrial influence of M_2_R-I_KACh_ has been well described [8–10], only recently have there been suggestions of an I_KACh_-like channel in the ventricle [11, 12, 16]. To investigate the influence of M_2_R-I_KACh_ on ventricular physiology, we utilized optical mapping of the LV, a technique which allows for the simultaneous examination of multiple characteristics of ventricular electrophysiology, such as APD, CV, APD heterogeneity *μ* and slope of APD restitution curve, *S*_*max*_.

### Action potential duration

**Fig 2:**
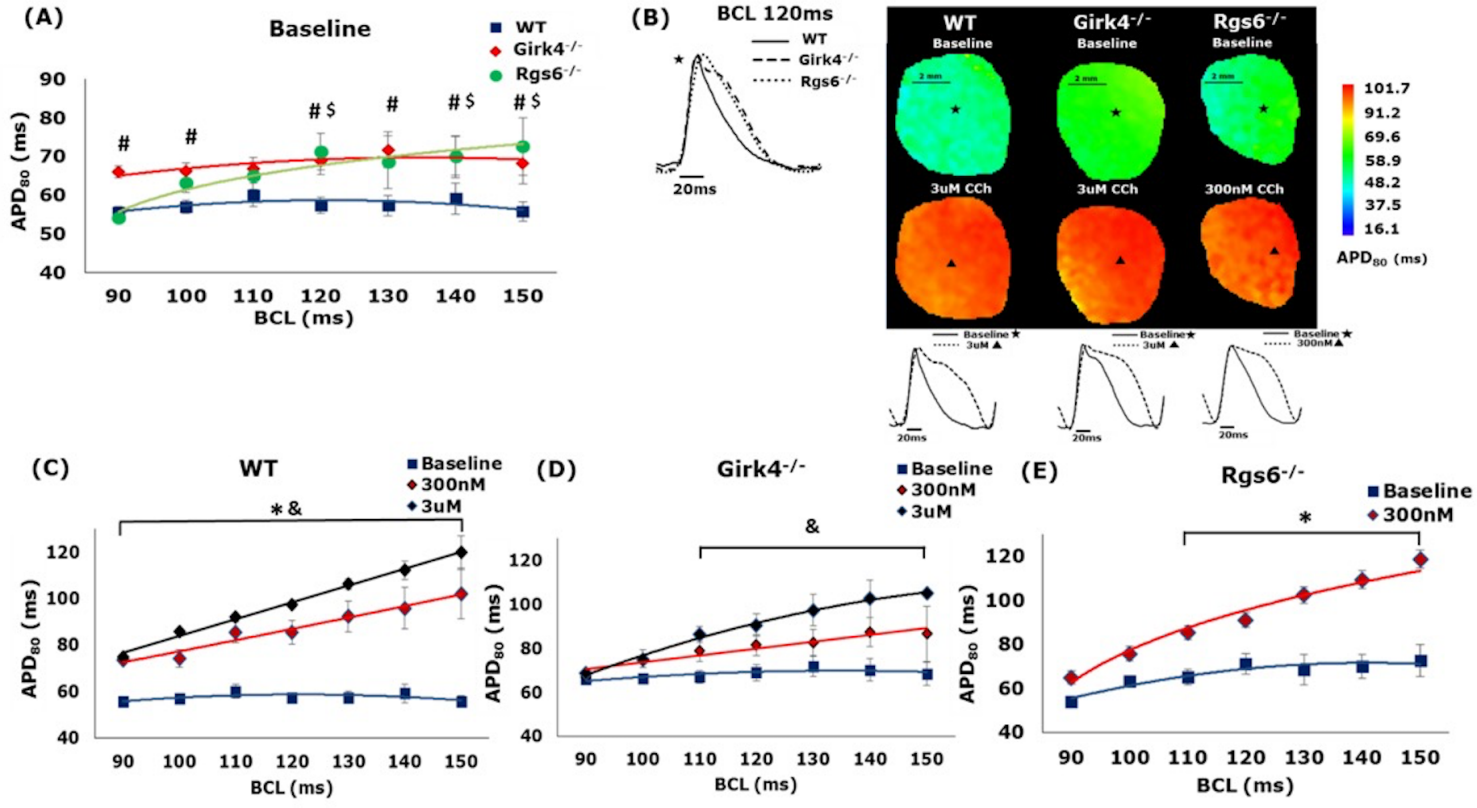
The effect of GIRK4 and RGS6 ablation on APD_80_. (A) Change in average APD_80_ with decreasing BCL in WT, *Girk4*^*-/-*^, and *Rgs6*^*-/-*^ hearts. (‘#’ denotes statistical significance of p < 0.05 between WT and *Girk4*^*-/-*^. ‘$’ denotes statistical significance of p < 0.05 between WT and *Rgs6*^*-/-*^). (B) Representative 2D APD_80_ maps from WT, *Girk4*^*-/-*^, and *Rgs6*^*-/-*^ hearts, constructed at BCL = 120 ms both at baseline and post-CCh injection. Representative action potential traces are shown at baseline (top panel, pixels marked by *) and post-CCh (bottom panel, pixels marked by Δ). (C-E) The effect of CCh on APD_80_ at decreasing BCL in WT, *Girk4*^*-/-*^, and *Rgs6*^*-/-*^ hearts. (‘*’ denotes statistical significance of p < 0.05 between baseline and 300nM CCh; ‘&’ denotes statistical significance of p < 0.05 between baseline and 3uM CCh). n = 8, 5, 8 for WT, *Rgs6*^*-/-*^, and *Girk4*^*-/-*^_,_ respectively. All statistics performed using 1-way ANOVA.

Optical movies taken from the LV were used to assess the impact of GIRK4 or RGS6 ablation on ventricular APD_80_. Notably, and consistent with the expected impact of K^+^ channels on repolarization, hearts from *Girk4*^*–/–*^mice showed a significant prolongation in baseline APD_80_ relative to WT controls at almost all BCLs (Fig 2A). Surprisingly, *Rgs6*^*-/-*^ mice also exhibited prolonged APD_80_, and this effect was significant only at higher BCLs (Fig 2A). Consistent with previous studies [16, 24, 25], CCh stimulation significantly increased APD_80_ across all genotypes and in a dose-dependent fashion (Fig 2C–2E). Since 3 μM CCh terminated beating in *Rgs6*^*-/-*^ mice, only the lower concentration of CCh (300 nM) was shown in these hearts. Collectively, these data suggest that while I_KACh_ impacts baseline ventricular APD in a predictable fashion, the influence of RGS6 is likely I_KACh_-independent. Furthermore, statistical analysis of the effect of CCh on APD across genotypes that was performed using 2-way ANOVA, did not reveal any significant differences. This suggests that although CCh significantly prolonged APD_80_ in all genotypes, the signaling pathway did not seem to meaningfully impact the effect of CCh on ventricular APD_80_.

### Slopes of APD restitution curve (*S*_*max*_)

**Fig 3:**
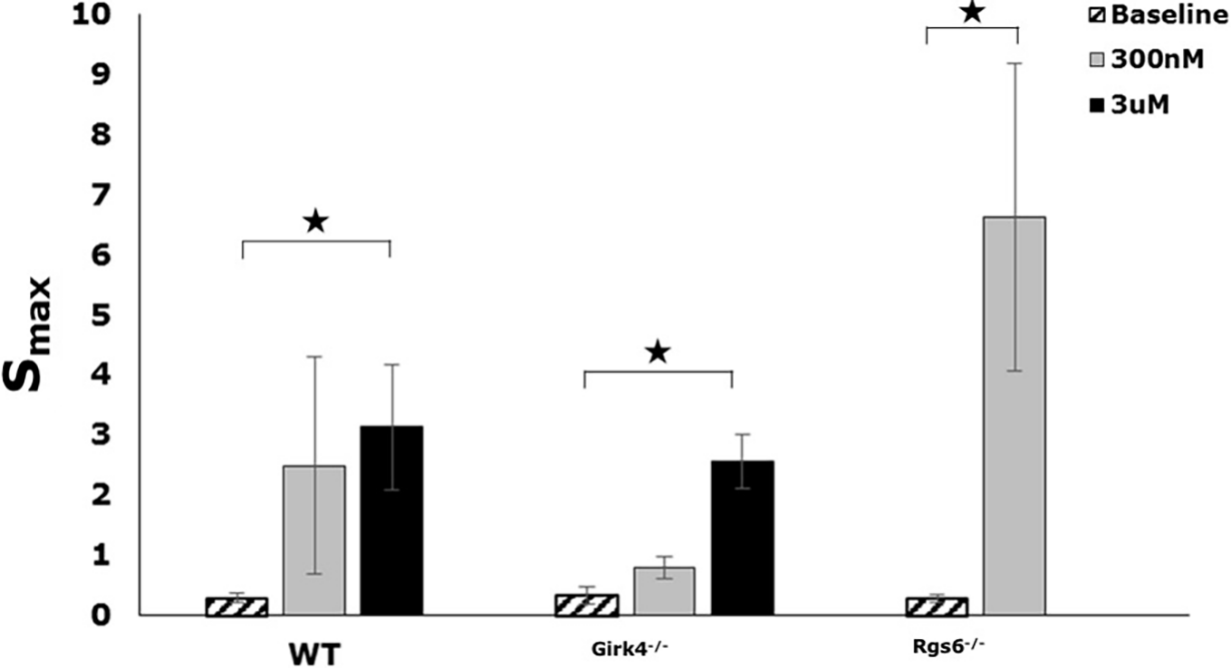
The effect of GIRK4 and RGS6 ablation on S_max_. Summary of the baseline and post-CCh maximum slopes of APD restitution, S_max_, in WT (n = 8), *Girk4*^*-/-*^ (n = 8) and *Rgs6*^*-/-*^ (n = 5) mice. (‘*’ denotes statistical significance of p < 0.05 between post-CCh and baseline for the same genotype. Statistics performed using 1-way ANOVA).

The slope of APD restitution curve describes the relationship between APD and preceding DI, and a steep maximum slope *S*_*max*_ > 1 is associated with an increase in arrhythmia susceptibility [26, 27]. Our results indicate that CCh administration yielded a *S*_*max*_ > 1 in all mice, indicating a pro-arrhythmic effect [26, 27], irrespective of genotype (Fig 3). In the case of WT and *Girk4*^*-/-*^ hearts, 3 μM CCh yielded a significantly larger *S*_*max*_, but there was no significant effect of 300 nM CCh (Fig 3). In contrast, hearts from *Rgs6*^*-/-*^ mice showed significant steepening of *S*_*max*_ at 300 nM CCh (Fig 3).

### CV and APD_80_ heterogeneity, μ

**Fig 4:**
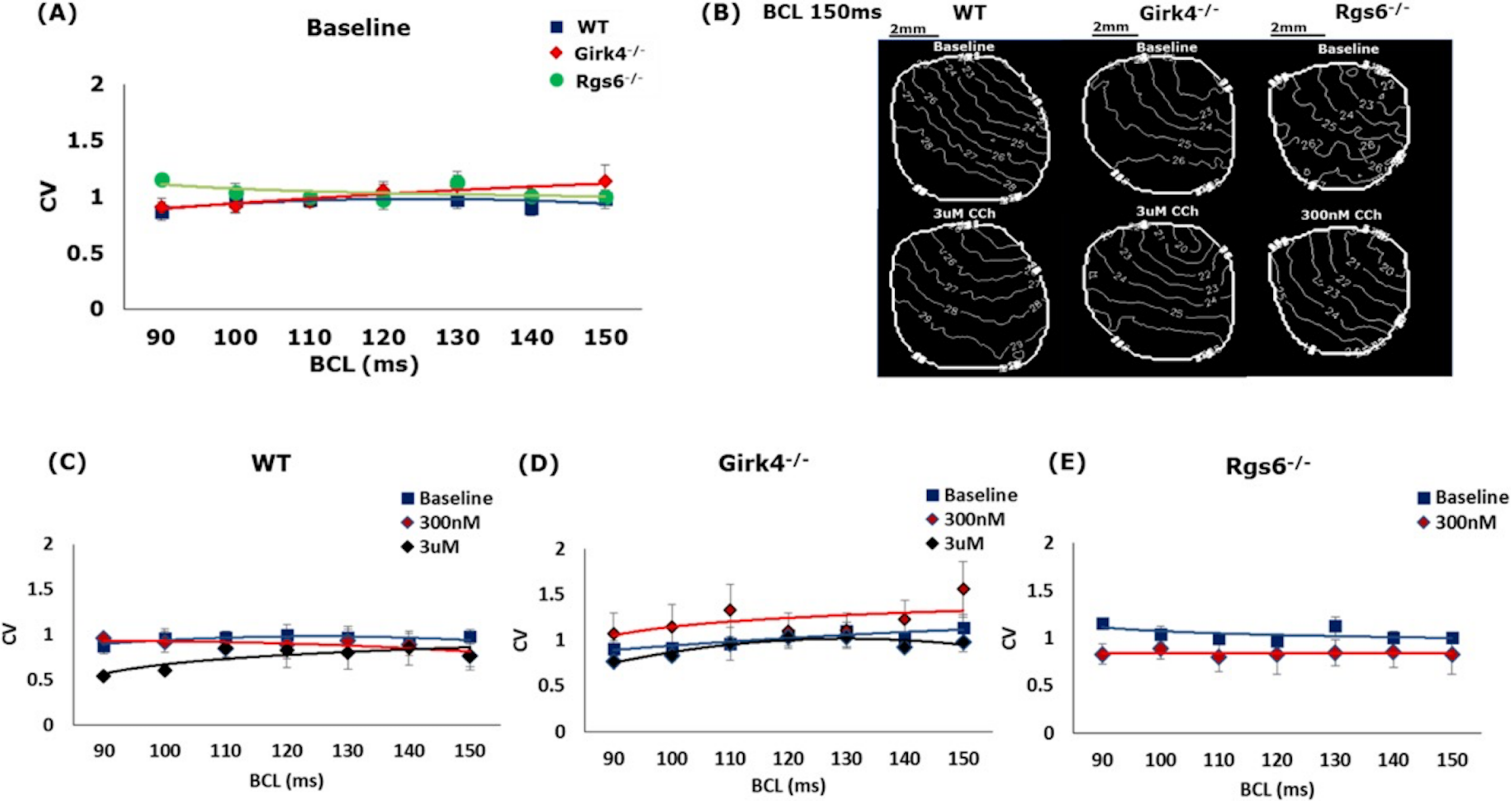
The effect of GIRK4 and RGS6 ablation on CV. (A) Average CV as a function of BCL in WT, *Girk4*^*-/-*^, and *Rgs6*^*-/-*^ hearts. (B) Representative 2D activation maps from WT, *Girk4*^*-/-*^, and *Rgs6*^*-/-*^ hearts, constructed at BCL = 150 ms both at baseline and post-CCh injection. (C-E) The effect of CCh on CV at different BCL in WT, *Girk4*^*-/-*^, and *Rgs6*^*-/-*^. n = 8, 5, 8 for WT, *Rgs6*^*-/-*^, and *Girk4*^*-/-*^_,_ respectively. Statistics performed using 1-way ANOVA.

**Fig 5:**
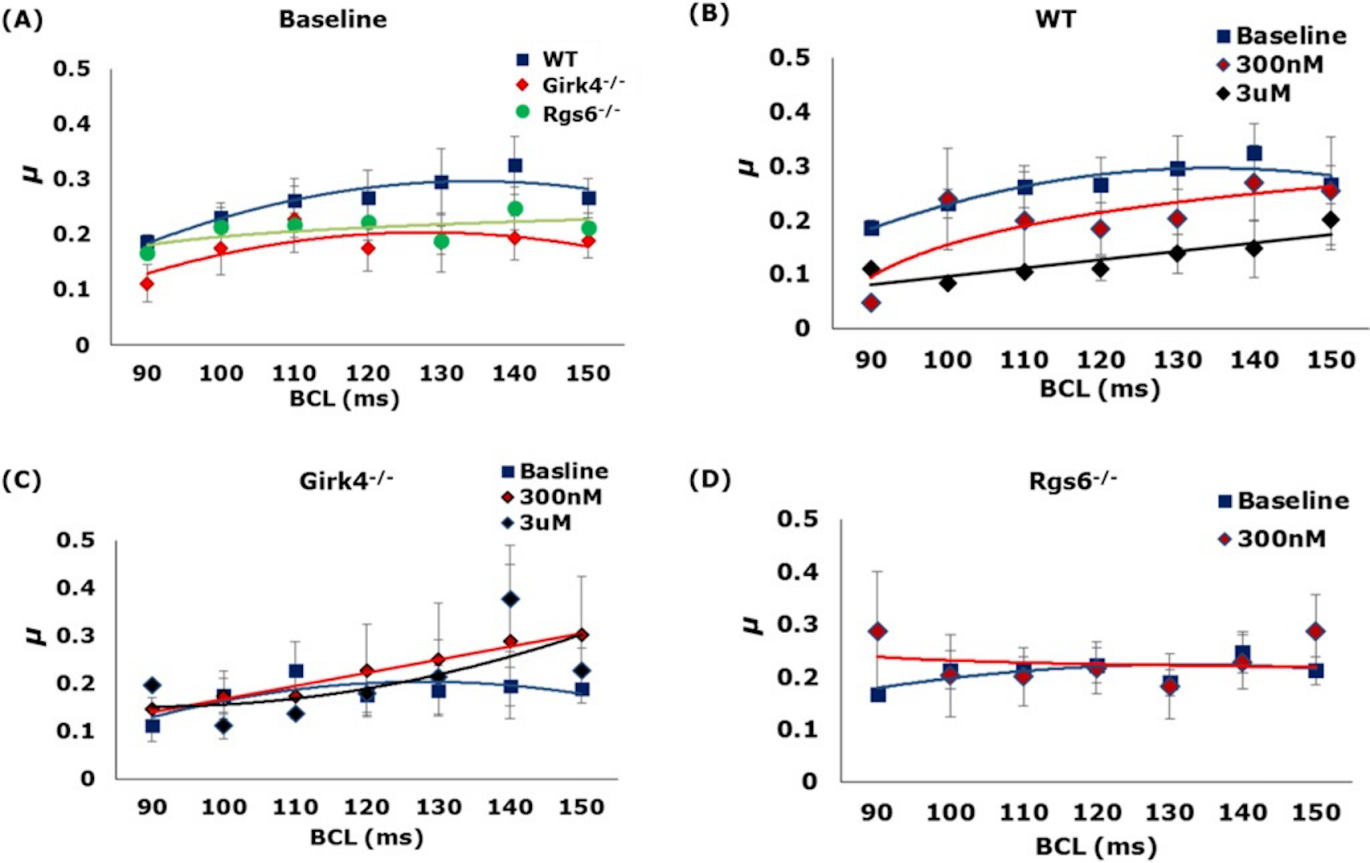
The effect of GIRK4 and RGS6 ablation on APD heterogeneity, μ. (A) Average APD heterogeneity, μ, as a function of BCL in WT, *Girk4*^*-/-*^, and *Rgs6*^*-/-*^ hearts. (B-D) The effect of CCh on μ at different BCL in WT, *Girk4*^*-/-*^, and *Rgs6*^*-/-*^ hearts. n = 8, 5, 8 for WT, *Rgs6*^*-/-*^, and *Girk4*^*-/-*^ respectively. Statistics performed using 1-way ANOVA.

Next, we measured the LV CV, a measurement of APD propagation across the ventricle, with decreasing BCL. We found that there was no impact of genotype on baseline CV values (Fig 4A). Similarly, no significant effect of CCh on LV CV was observed in any of the genotypes (Fig 4C–4E). Representative activation maps demonstrate smooth propagation of action potentials for all the three groups both at baseline and post CCh (Fig 4B). The change in LV APD_80_ heterogeneity, *μ*, was also measured, given the critical role heterogeneity plays in arrhythmogenesis. Again, there were no significant differences in APD heterogeneity observed between the three mice groups, both at baseline and post injection of CCh (Fig 5A–5D).

## Discussion

In this study, we investigated the impact of the M_2_R-I_KACh_ signaling pathway on ventricular electrophysiology using both *Girk4*^*–/–*^and *Rgs6*^*–/–*^mice. There is a body of published evidence supporting the contention that a GIRK4-containing GIRK channel is expressed in the mouse ventricle, and that it mediates the effects of cholinergic input on ventricular myocyte excitability [11, 12]. Our current work supports a critical role for this signaling pathway on the parasympathetic regulation of HR and HRV, while also suggesting intriguing differences in the roles of key pathway elements on the parasympathetic regulation of ventricular physiology. Indeed, our efforts support the emerging perspective that RGS6 impacts multiple electrophysiological parameters in the ventricle, likely apart from any influence it has on signaling involving a ventricular I_KACh_-like channel.

Our major findings are as follows: (1) Ablation of Girk4 and RGS6 can lead to electrophysiological remodeling and affect overall cardiac dynamics. (2) Parasympathetic stimulation of *Girk4*^*-/-*^ and *Rgs6*^*-/-*^ mice leads to a dose-dependent prolongation in APD_80_ and steepening of APD restitution. (3) While the M_2_R-Girk4-RGS6 pathway plays an important role in regulating cardiac dynamics, the effects of parasympathetic stimulation of the ventricles could be mediated via M_2_R-I_KACh_ independent pathways.

### *In-vivo* ECG

Our *in-vivo* ECG data confirmed many previously reported phenotypes of *Girk4*^*-/-*^ and *Rgs6*^*-/-*^ mice, highlighting the opposing influences that RGS6 and I_KACh_ exert on atrial physiology. *Rgs6*^*-/-*^ mice displayed significantly lower baseline HR as well as exaggerated responses to CCh-induced bradycardia and increases in HRV, all of which have been previously reported [5–11]. Conversely, *Girk4*^*-/-*^ mice displayed blunted responses to the impact of CCh on HR and HRV. While the baseline HR in the *Girk4*^*-/-*^ mice was higher than that of the WT mice, this effect was not significant. Lastly, we also show that *Rgs6*^*-/-*^ mice were more susceptible to CCh-induced arrhythmic episodes, whereas *Girk4*^*-/-*^ mice were resistant. These data further emphasize the contrasting roles I_KACh_ and RGS6 play in mediating parasympathetic influence on atrial physiology.

### *Ex-vivo* results

To characterize the potential influence of I_KACh_ and RGS6 on ventricular electrophysiology, we employed, for the first time, a high resolution optical mapping of the ventricle to analyze 2D APD_80_, CV and APD heterogeneity. As expected, we observed a prolongation in APD_80_ at baseline in *Girk4*^*-/-*^ mice, consistent with previous reports and the presence of a functional I_KACh_-like channel in ventricular myocytes [11]. Although previous studies have investigated the effect of RGS6 ablation on atrial APD [9, 10], its role and impact on ventricular electrophysiology is relatively unknown. This is the first study to investigate the effect of RGS6 ablation on APD restitution and characterize its effect on ventricular APD at different pacing rates. Surprisingly, hearts from *Rgs6*^*-/-*^ mice also showed a prolongation in APD_80_, particularly at higher BCLs. Our results strongly indicate that RGS6 does play an important role in ventricular repolarization, while also arguing that this influence is not mediated via its influence on a ventricular I_KACh_-like channel. This is also supported by the fact that at 300 nM of CCh, there was a significant increase in the slope of APD restitution in *Rgs6*^*-/-*^ mice, but no difference between WT and *Girk4*^*–/–*^mice. Given the lack of impact GIRK4 ablation had on this parameter, this further suggests an I_KACh_-independent mechanism for RGS6 influence in mediating ventricular physiology. Indeed, RGS6 has been shown to impact cardiac physiology independent of its GTPase activity via reactive oxygen species (ROS)-dependent mechanisms [28–30]. Specifically, RGS6 mediates doxorubicin-induced cardiotoxicity through a ROS mechanism. Furthermore, it has also been reported that loss of RGS6 predisposes the ventricle to pro-death signaling through a β2 Adrenergic and G protein-coupled receptor kinase 2-dependent signaling mechanism [29]. Hence, it is possible that the pronounced effect of CCh in *Rgs6*^*-/-*^ mice could be the result of indirect effects of parasympathetic stimulation or accentuated antagonism.

CCh increased APD_80_ in hearts from WT, *Girk4*^*–/–*^, and *Rgs6*^*-/-*^ mice, consistent with previously reported effects of vagal nerve stimulation (VNS) on ventricular APD. The APD_80_ after CCh injection, at both 300 nM and 3 μM, was not significantly different across all genotypes. This indicates that M_2_R-I_KACh_ signaling does not significantly mediate the influence of CCh on APD_80_. This is also supported by the fact that there was no difference in the CCh increase in the slopes of APD restitution between WT and *Girk4*^*–/–*^mice. CCh did increase the slope of APD restitution in *Rgs6*^*-/-*^ mice at a lower concentration than WT and *Girk4*^*–/–*^mice. This suggests an I_KACh_-independent mechanism for RGS6 influence in mediating ventricular physiology. Indeed, supporting our current finding, it has been shown previously that the anti-arrhythmic effects of vagal stimulation can occur via nitric oxide, independent of muscarinic receptor activation [24, 25]. Additionally, there was no significant effect of either GIRK4 or RGS6 ablation on CV or APD heterogeneity. Since CV is predominantly affected by sodium channel activity, a lack of significant effect of I_KACh_ on ventricular conduction was not surprising.

Recently, significant interest has been placed on understanding the molecular mechanisms underlying vagal input to the ventricles given innovative investigations into the efficacy of VNS as a therapy for metabolic and cardiovascular diseases. Multiple studies have reported beneficial effects of VNS in restoring autonomic imbalance by parasympathetic activation of the healthy [31] and diseased [32–35] hearts. One previous study [31] suggests that chronic VNS leads to beneficial electrophysiological remodeling of the heart and therefore has a potential to treat cardiovascular diseases such as hypertension and myocardial ischemia. Therefore, a full understanding of the molecular mechanisms underlying vagal effects on cardiac physiology, which include M_2_R-I_KACh_ signaling, is necessary. In that regard, this study contributes to understanding the effects of parasympathetic modulation on ventricular electrophysiology.

## Limitations

Since the *in-vivo* ECGs in this study were acquired under anesthesia, baseline HRs were much lower than what is observed in conscious mice (Fig 1A) [36]. However, similar quantities of anesthesia were administered for all mice and effect of CCh was compared to baseline recordings in the same animal with no change in anesthesia administration during the experiment. In addition, a 20-minute stabilization window was used for each mouse prior to recording baseline HR, to enable the effect of anesthesia to stabilize. Hence, although the overall lower HRs could be attributed to the effect of anesthesia in each mouse [37] the differences in HRs observed can be attributed to the effect of genotype and CCh administration. Furthermore, the current study utilizes knock out mice with GIRK4 or RGS6 completely ablated in both the atria and ventricles. Future studies could be performed utilizing a pharmacological I_KACh_ blocker to investigate the relative importance of this pathway under varying conditions. In addition, ventricle and atria specific GIRK4 or RGS6 knock out models could be utilized to isolate the electrophysiological effects observed, to atrial or ventricular signaling pathways.
