# Correction: The influences of the M_2_R-GIRK4-RGS6 dependent parasympathetic pathway on electrophysiological properties of the mouse heart

**DOI:** 10.1371/journal.pone.0200553

**Published:** 2018-07-06

**Authors:** Kanchan Kulkarni, Xueyi Xie, Ezequiel Marron Fernandez de Velasco, Allison Anderson, Kirill A. Martemyanov, Kevin Wickman, Elena G. Tolkacheva

The third author's last name appears incorrectly in the citation. The correct citation is: Kulkarni K, Xie X, Marron Fernandez de Velasco E, Anderson A, Martemyanov KA, Wickman K, et al. (2018) The influences of the M2R-GIRK4-RGS6 dependent parasympathetic pathway on electrophysiological properties of the mouse heart. PLoS ONE 13(4): e0193798. https://doi.org/10.1371/journal.pone.0193798
